# Personal Log, Stardate 42552.6

**DOI:** 10.3201/eid1602.AD1602

**Published:** 2010-02

**Authors:** Curi Kim

**Affiliations:** Centers for Disease Control and Prevention, Detroit, Michigan, USA

**Keywords:** tuberculosis, measles, influenza, coxsackievirus, communicable diseases, parasites, epidemiology, another dimension

Awaiting the alien delegation from Sarona VIII
To scan them for communicable space disease,
I think of history and can’t help but now speculate
If our ancestral officers in duty were at ease
At their own quarantining stations, earthbound ports of entry,
There watchful for all ancient scourges crossing borders
(tuberculosis … measles … influenza … coxsackie),
Assessing travelers sans medical tricorders,
But using only judgment and with epi skills equip’d
To help determine meaning in a cough, a rash;
And knowing what they saw was barely the berg’s tip
Considering all the infected travelers they didn’t “catch”—
Ah, the delegates just beamed aboard to be met;
Let’s see what interstellar parasites we detect!

